# Genomic and Morphological Evidence Support Contemporary Three‐Way Interspecific Hybridization in Ranid Frogs

**DOI:** 10.1002/ece3.72035

**Published:** 2025-08-31

**Authors:** Owen M. Edwards, Neil R. Balchan, Kaleb M. Banks, Bo Zhang, Fabio A. Machado, Michael S. Reichert, Damien Esquerré

**Affiliations:** ^1^ Department of Biology Oklahoma State University Stillwater Oklahoma USA; ^2^ Oklahoma Biological Survey University of Oklahoma Norman Oklahoma USA; ^3^ Environmental Futures Research Centre, School of Science University of Wollongong Wollongong New South Wales Australia

**Keywords:** cranial morphometrics, introgression, *Lithobates*, *Rana areolata*, *Rana palustris*, *Rana sphenocephala*

## Abstract

Hybridization is increasingly understood as common throughout and beyond the speciation process, rather than an anomaly. Sympatric taxa are expected to exhibit strong reproductive isolation, and although hybridization may occur, it often results in inviable offspring. We investigated hybridization among three ranid frogs ( *Rana areolata* , *R. palustris*, and *R. sphenocephala* ) in eastern Oklahoma, where their distributions and breeding phenology overlap. Using micro‐CT scans of cranial morphology, genomic SNP data, and phenological records, we confirmed two putative hybrids collected in the field—remarkable given the phylogenetic distance between these non‐sister taxa. Genomic data show split ancestry from parental populations, representing one *R. areolata* × *R. palustris* and one *R. areolata* × *R. sphenocephala* . Cranial morphology indicates hybrids exhibit intermediate phenotypes, and our assessment identified a third likely hybrid, a specimen collected two decades earlier from the same area. Both confirmed hybrids were *R. areolata* backcrosses, but minimal introgression throughout the dataset suggests hybrid fitness may be lower than that of parental populations. Hybridization appears facilitated by overlapping breeding strategies and ecological factors leading to misdirected amplexus. This study provides the first documentation of natural hybridization in *R. areolata* , a species of conservation concern throughout its range. Our findings emphasize the utility of high‐resolution morphological data (micro‐CT) in complementing genomic approaches for hybrid diagnosis and suggest cranial morphology may be an effective method for hybrid identification in similar systems. Understanding this atypical three‐species hybridization has important implications for conservation, as hybrid fitness and introgression can influence population dynamics and genetic integrity.

## Graphical abstract

We investigated natural hybridization among three rapid frogs in eastern Oklahoma. Using genomic SNP data and cranial morphometrics, we confirmed the presence of hybrids exhibiting intermediate traits. Despite overlapping breeding phenologies, minimal introgression suggests hybrid fitness may be considerably lower than that of parental populations.

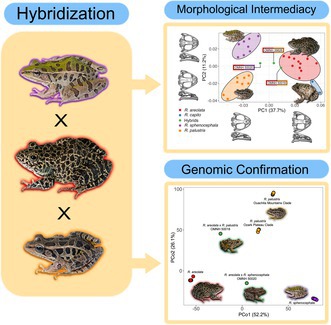

## 1 Introduction

Hybridization, the successful breeding between species, has traditionally been seen as a rare event that produces inviable offspring and is used as the ultimate test for the Biological Species Concept (De Queiroz 2007; Hillis 2019). However, in recent years, it has been shown that interspecific hybridization is more common than previously thought across the tree of life (Taylor and Larson 2019; McEntee et al. 2020; Peñalba et al. 2024) and that it serves as an evolutionary mechanism that facilitates adaptive introgression (Gaczorek et al. 2024), enhances genetic diversity, and even leads to the formation of new species (Mikkelsen and Weir 2023). In allopatric speciation, spatial separation of populations facilitates divergence, but when speciation occurs between taxa in sympatry, this divergence may be driven by behavioral, ecological, or life history mechanisms (Bolnick and Fitzpatrick 2007). These processes lead to the accumulation of reproductive isolation mechanisms (both pre‐ and post‐zygotic) that function to maintain the distinction of the lineages even upon secondary contact (Pulido‐Santacruz et al. 2018). However, reproductive isolation is not always complete, and hybridization may occur, with potential fitness consequences (positive or negative) for hybrid individuals.

Secondary contact of previously allopatric lineages may allow for hybridization in relatively narrow contact zones at historic (Devitt et al. 2011) or contemporary time points (Maag et al. 2023). When strong pre‐ or postzygotic barriers exist, hybridization in these zones may be nonexistent or limited (Cowles and Uy 2019); but taxa with weaker barriers may see considerable amounts of hybridization occurring in contact zones (Zancolli et al. 2016). In anurans, zones of secondary contact (e.g., Loftus‐Hills and Littlejohn 1992; Green and Parent 2003; Smith et al. 2013) can result in admixed individuals with variable and asymmetric introgression patterns of parental genomes (Majtyka et al. 2022).

Conversely, taxa co‐occurring over large areas of their distributions should be expected to hybridize less frequently, as strong reproductive barriers are expected to maintain genetic segregation of lineages (Coyne and Orr 1989; Howard 1993; Butlin 1998). Despite this expectation, cases of interspecific hybridization among broadly co‐occurring species are reported frequently (e.g., Tubaro and Lijtmaer 2002), though these hybrids may be of limited evolutionary consequence given their sporadic generation and maladapted nature in comparison to parental populations (Hoskin and Higgie 2013; Pärssinen et al. 2020). Anurans serve as ideal systems for exploring hybridization because of their reproductive traits (i.e., external fertilization; Lantiegne and Purchase 2023), and various organismal facets including genetics (Austin et al. 2011), morphology (Schlefer et al. 1986), and acoustics (Lemmon and Juenger 2017) have the potential to display strong interspecific differences from which intermediate forms can be readily identified. Anuran species co‐occurring over large areas may hybridize because of unique aspects of their breeding ecology, such as multi‐species reproductive aggregations, where mate signaling and breeding occur in a shared body of water at the same time. These mass reproductive events often lead to heterospecific amplexus (i.e., misdirected amplexus; Serrano et al. 2022), which may be most prevalent in explosive breeding species where male scramble competition is increased at the expense of mate choice/selectivity (Wells 1977). Misdirected amplexus (and subsequent hybridization) is thought to be much less common among frogs with extended breeding seasons (Gerhardt et al. 1994; Simões et al. 2012), especially in species with intricate courtship behaviors (e.g., multimodal signaling), where close interactions between signalers and receivers likely function as pre‐mating isolation mechanisms (Wells 2007; Nali et al. 2023).

Additional complexity may arise when more than two lineages share breeding areas, potentially resulting in atypical genetic exchange dynamics, including three‐way hybridization and triad hybridization, whereby an intermediary species facilitates gene flow between two additional taxa (Grant and Grant 2020). Hybrid viability and the potential for backcrossing are vital for allowing these complex dynamics to be maintained over time. Instances of hybridization among three species have been documented in a variety of taxonomic groups (Crow et al. 2007; Fontaine et al. 2015; Haines et al. 2016), yet they remain poorly characterized. Documenting and understanding the evolutionary consequences of these tri‐species hybridization systems is crucial, as they may generate novel or complex genetic combinations, potentially accelerating genetic exchange and adaptive introgression among diverged lineages.

**FIGURE 1:**
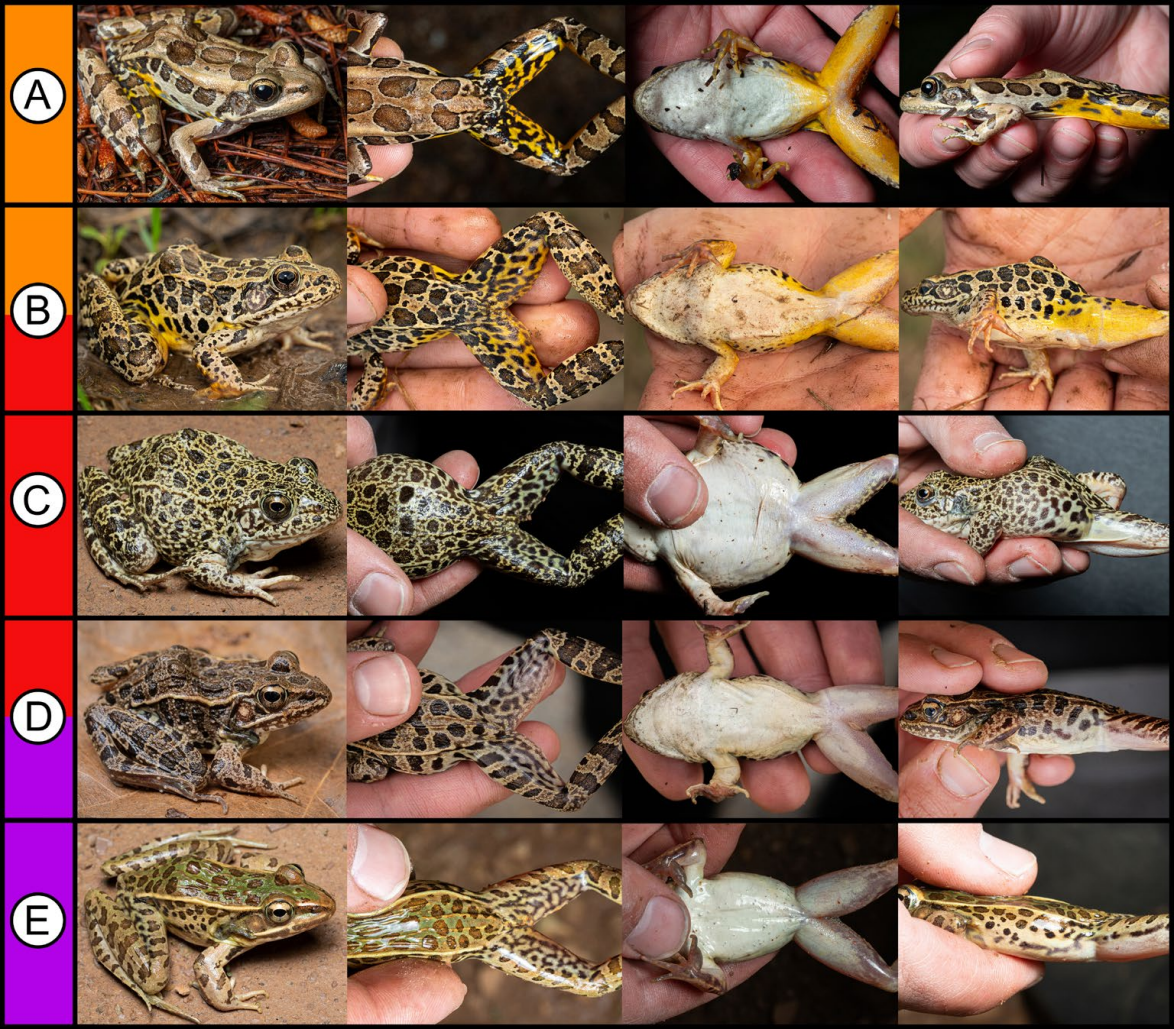
Photographs in life depicting pure individual body orientations of (A) the pickerel frog ( *Rana palustris* ), (C) crawfish frog ( *R. areolata* ), and (E) southern leopard frog (*R. sphenocephala*) originating from the Atoka County field site. Photographs of hybrids depict general profile, dorsal, ventral, and lateral aspects of (B) female *R. areolata* × *R. palustris* (OMNH 50018) and (D) male *R. areolata* × *R. sphenocephala* (OMNH 50020). In (B), note the yellow venter and block‐shaped spots of OMNH 50018 (consistent with *R. palustris* ) and in (D) the exaggerated dorsolateral folds of OMNH 50020 (consistent with *R. sphenocephala* ).

**FIGURE 2:**
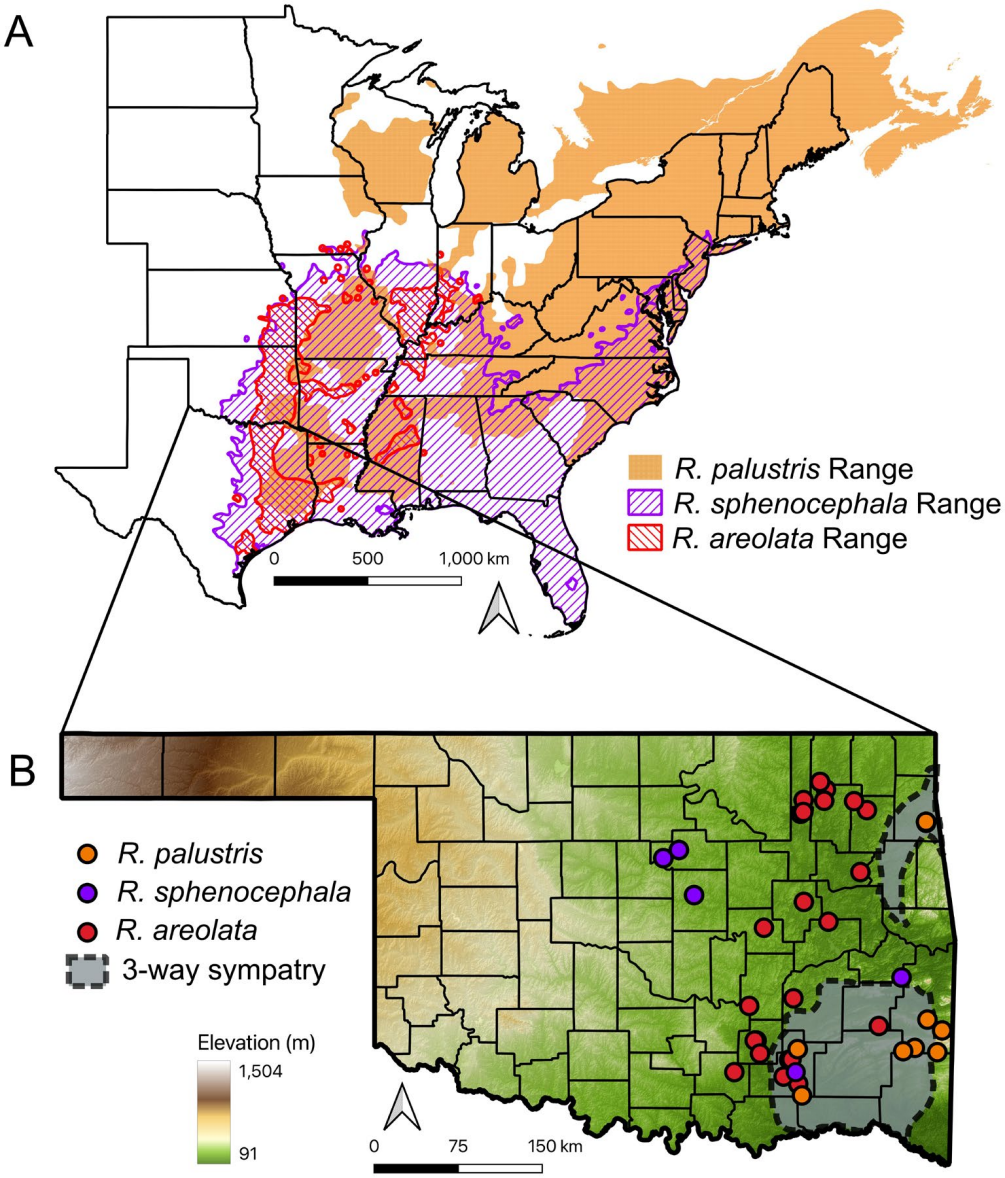
(A) Partial map of the central and eastern United States displaying the geographic distributions on the basis of Powell et al. (2016) for the pickerel frog ( *Rana palustris* ), southern leopard frog ( *R. sphenocephala* ), and crawfish frog ( *R. areolata* ). (B) Map of the state of Oklahoma depicting the spatial sampling of tissues collected in this study and the area of three‐way sympatry among the three focal taxa. Points on the map may represent multiple individuals sampled at the same locality.

In the present study, we examined hybridization dynamics among three syntopic ranids (genus *Rana*, *sensu* Yuan et al. 2016; Figure 1A,C,E) in southeastern Oklahoma, USA: the crawfish frog ( *Rana areolata* ), pickerel frog ( *R. palustris* ), and southern leopard frog ( *R. sphenocephala* ), which are highly divergent taxa (Hillis and Wilcox 2005; Yuan et al. 2016) that differ from one another considerably in acoustic signals (Lannoo et al. 2018), behavior, and morphology (Engbrecht et al. 2011). *Rana areolata* and *R. palustris* are both members of the subgenus *Nenirana* (Hillis and Wilcox 2005), a four‐species clade including two additional taxa: the gopher frog ( *R. capito* ) and dusky gopher frog ( *R. sevosa* ). *Nenirana* are ecologically united by their dependence on robust subterranean shelters during non‐breeding periods (Engbrecht et al. 2011), and two species ( *R. capito* [Vulnerable] and *R. sevosa* [Critically Endangered]) are of major conservation concern (Richter et al. 2014; Hinkson and Richter 2016). *Rana areolata* has recently experienced significant population‐level declines throughout its range primarily because of habitat loss and landscape changes (e.g., Engbrecht et al. 2012; Lannoo and Stiles 2020; Kross and Willson 2022; Boycott et al. 2024) and is subsequently of conservation concern in every state in which it occurs (Lannoo and Stiles 2020). *Rana areolata* is unique in its obligate commensal relationship with crayfish (Decapoda: Astacoidea), whereby it inhabits abandoned crayfish burrows for the entirety of its non‐breeding period (Heemeyer et al. 2012). The brief breeding window of this species, coupled with its fossorial nature, has resulted in *R. areolata* being considered one of the most secretive anurans in North America (Engbrecht and Lannoo 2012; Lannoo and Stiles 2020). *Rana sphenocephala* differs in belonging to the clade *Scurrilirana*, a group in which all species share distinctive “chuckle‐like” advertisement calls (Hillis and Wilcox 2005), but do not have major constraints on life history as in *Nenirana*. These three ranid species have wide‐ranging distributions in the United States, with vast areas of sympatry (Figure 2).

Breeding experiments in a laboratory setting indicate that many *Rana* species will readily hybridize (Hillis 1988), with F1 offspring displaying variable levels of hybrid inviability. Lab crosses conducted with female *R. areolata* against male *R. palustris* and *R. sphenocephala* resulted in viable offspring that successfully developed to metamorphosis without evidence of developmental abnormality (Mecham 1969; Cuellar 1971). Despite examples of experimental hybridizations and widespread sympatry among these three species, there is no evidence that hybridization occurs naturally; yet the possibility of hybridization exists because of the unique behavioral and ecological context of this system.

Here, we use cranial morphometrics and genomic data to address hybridization in this system, hypothesizing that morphological and genomic analyses would confirm mixed ancestry in putative hybrids observed during recent population monitoring (Edwards et al. 2025). Additionally, we explored if widespread introgression exists among species, if hybrids represent F1 or backcrossed individuals, and if morphological intermediacy can diagnose hybrids.

## 2 Methods

### 2.1 Fieldwork and Sampling

*Rana areolata* population monitoring was conducted at an abandoned cattle pond (~247 m in circumference) in Atoka Public Hunting Area (PHA; 34.54102, −95.93999) in Atoka County, Oklahoma, USA. From 13 February 2023 to 4 April 2023, we captured all anurans entering and exiting the pond with the use of drift fences paired with pitfall traps (see Edwards et al. 2025 for more details). The drift fences and pitfall traps were monitored at least twice daily, once after sunrise and once after sunset. During nights with intense chorusing activity or large rain events, we increased the frequency of drift fence checks, continuing until no additional anurans were observed. Captured *R. areolata* were weighed to the nearest 0.1 g, measured for snout‐vent length (SVL) and tibia‐fibula length to the nearest 0.1 mm, and sexed by checking for the presence of enlarged vocal sacs associated with breeding males. Newly captured *R. areolata* received a unique passive integrated transponder (PIT) tag, were photographed, and toe‐clipped for genetic tissue samples (*n* = 19 used herein). After processing, *R. areolata* were released at the opposite side of the fence from where they were captured. All other anurans were visually identified to species and sex and released accordingly.

During population monitoring in 2023, we discovered two putative hybrids, tentatively identifying one individual as a female *R. areolata* × *R. palustris* (OMNH 50018) and the other as a male *R. areolata* × *R. sphenocephala* (OMNH 50020) because of intermediate dorsal and lateral patterns and atypical body proportions (Figure 1). Additional examination of fluid‐preserved specimens at the Sam Noble Oklahoma Museum of Natural History (OMNH) revealed a third putative hybrid resembling a female *R. areolata* × *R. palustris* (Appendix 1) from the vicinity of our study site that was collected in 2003 (OMNH 39826).

Voucher specimens with liver tissue samples (in 100% ethanol) were collected from individuals at the study pond: *R. sphenocephala* (*n* = 10), *R. palustris* (*n* = 7), *R. areolata* × *R. palustris* (*n* = 1), and *R. areolata* × *R. sphenocephala* (*n* = 1). We collected voucher specimens of *R. sphenocephala* (*n* = 20) from an additional four populations (Figure 2) where the species exists in allopatry from the other two taxa and took liver tissue or tail clippings in 100% ethanol. Additional tissue samples (liver in 95% ethanol) of *R. palustris* from two populations (*n* = 10) were loaned from OMNH.

### 2.2 Micro‐Computed Tomography (Micro‐CT) and Geometric Morphometrics

**FIGURE 3:**
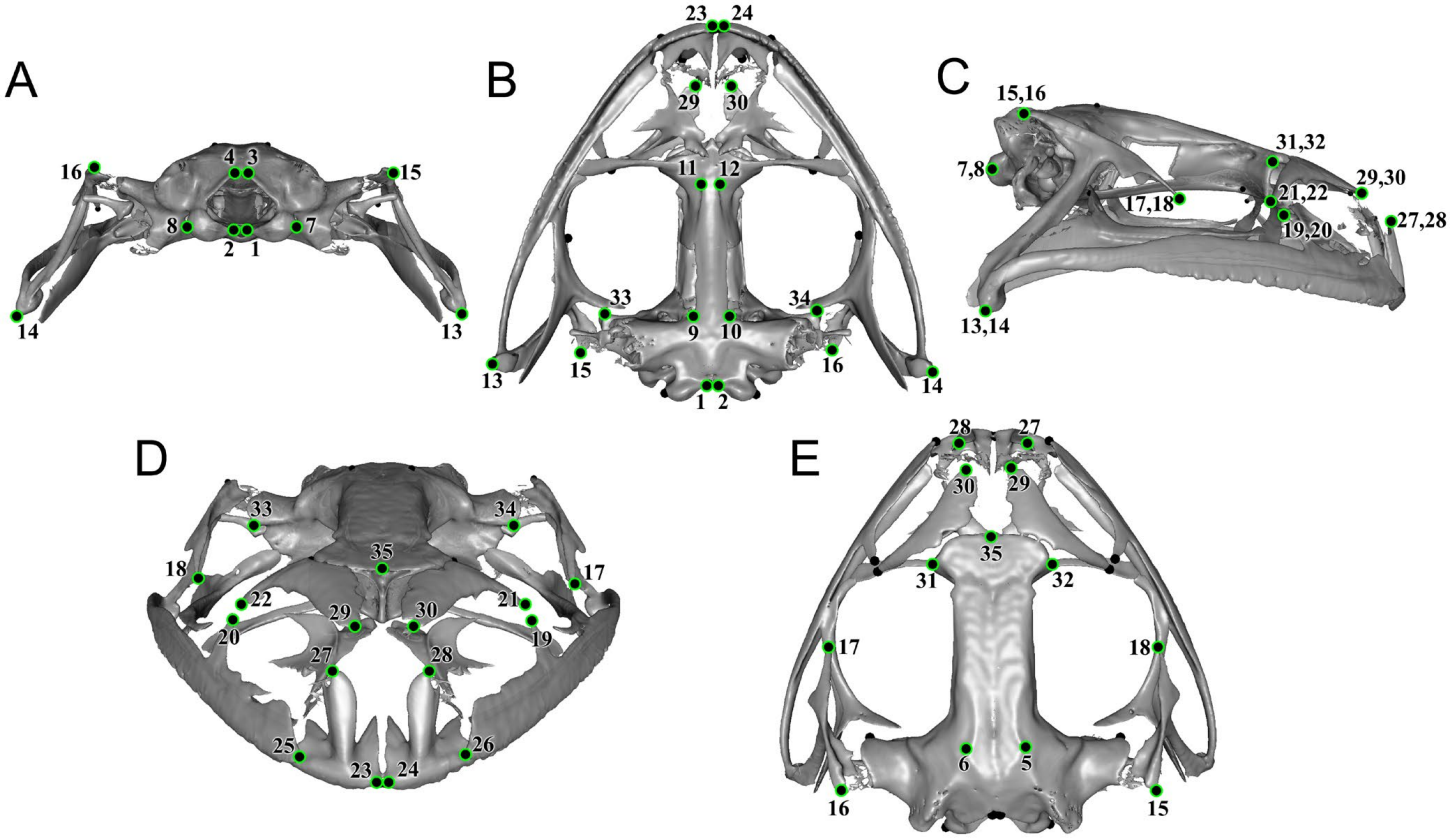
Location of 35 landmarks placed onto each cranial scan ( *Rana areolata* , OMNH 50032 shown here), viewed from (A) posterior, (B) ventral, (C) lateral, (D) anterior, and (E) dorsal aspects.

Cranial morphology data were obtained from 32 fluid‐preserved specimens (*n* = 11 *R. areolata* ; *n* = 8 *R. palustris* ; *n* = 8 *R. sphenocephala* ; *n* = 3 hybrids [*n* = 1 *R. areolata* × *R. sphenocephala* and *n* = 2 *R. areolata* × *R. palustris* ]; and *n* = 2 *R. capito* ). We included *R. capito* because this species is closely related to both *R. areolata* and *R. palustris* , and is known to possess unique skull morphology (Engbrecht et al. 2011). All specimens (apart from *R. capito* ) originated from Atoka County, Oklahoma, to control for geographic variation (Appendix 2). Specimens were scanned with a Carl Zeiss Xradia Versa XRM 410 micro‐computed tomography system (MicroCT; Carl Zeiss X‐Ray Microscopy Inc., Pleasanton, California, USA) at the Oklahoma State University Advanced Technology Research Center, with X‐ray source voltage and current set to 40 kV and 250 mA, respectively. Scans were performed with a resolution of 32.41 μm, and raw tomography files for each specimen were manually reconstructed with the ZEISS scout‐and‐scan Control System Reconstructor program. Visualization, volume rendering, and segmentation were all performed using 3D Slicer software 5.4.0 (Fedorov et al. 2012) and the SlicerMorph extension (Rolfe et al. 2021). Thirty‐five anatomical landmarks (one on the midline and 17 symmetrically positioned on each side of the skull; Figure 3) were manually digitized onto each cranial model. A generalized Procrustes analysis (GPA; Rohlf and Slice 1990) was conducted using the package Morpho version 2.12 (Schlager 2017) to translate, rotate, and scale cranial landmarks to a common coordinate system. The superimposed configurations were then projected onto a tangent Euclidean space (Slice 2001) and the covariance matrix of Procrustes residuals (representing the differences between the mean shape and each observed shape) was subjected to principal component analysis (PCA). Two PCA axes were used to visualize cranial shape variation among species and hybrids. To statistically assess overall skull morphological differences among species groups, we excluded hybrid individuals and performed a non‐parametric multivariate analysis of variance (np‐MANOVA; Anderson 2001) on the principal component scores using the “adonis2” function from the vegan package version 2.6–8 (Dixon 2003). The np‐MANOVA's *F* statistic and associated level of significance were calculated with 999 permutations. We deposited image stacks (TIFF) and 3D mesh files (STL) in MorphoSource (Project ID: 000720563).

To evaluate the intermediate morphological characters of hybrids, we performed a linear discriminant analysis (LDA) using the MASS package version 7.3–60 (Venables and Ripley 2002). LDA identifies axes of variation, called discriminant functions, that maximize the separation between predefined groups by maximizing the ratio of between‐group to within‐group variance (Campbell and Atchley 1981). To ensure unbiased classification, hybrids and *R. capito* were excluded from the initial training dataset used in the LDA model. We also applied a jackknife (“leave‐one‐out”) cross‐validation (LCV) to evaluate the robustness of classification accuracy and group superposition of the LDA (Machado and Hingst‐Zaher 2009). In this approach, each specimen is sequentially excluded from the dataset, and the LDA model is trained on the remaining individuals to generate the discriminant function. The excluded specimen is then classified on the basis of this model, and the process is repeated for every individual in the dataset. This procedure yields an average classification error and calculates a probability of group membership for each individual. For each individual hybrid, we calculated posterior probabilities of membership in each pure species group, quantifying their morphological similarity to parental species and allowing us to assess the probability of hybrids belonging to a single parental group.

### 2.3 Molecular Sampling and SNP Filtering

We gathered 94 tissue samples (Appendix 2) from the three species of *Rana* and two putative hybrids: *R. areolata* (*n* = 45), *R. palustris* (*n* = 17), *R. sphenocephala* (*n* = 30), and hybrids (*n* = 2). Several of these *R. sphenocephala* (four populations; *n* = 20) were selected because they came from areas of allopatry with *R. areolata* and thus represent pure populations. We genotyped samples using the DArTseq (Diversity Array Technology Sequencing) platform designed by Diversity Arrays Technology, Canberra, Australia (DArT). This genome complexity reduction technique combines fragmenting by restriction enzymes, filtering by fragment size, and sequencing (Georges et al. 2018; Kilian et al. 2012) to generate data that include thousands of fragments associated with restriction sites and their accompanying single nucleotide polymorphisms (SNPs), and such data have proven useful to evaluate phylogeographic structure and species limits (Chaplin et al. 2019; Esquerré et al. 2020; Georges et al. 2018). The DArT bioinformatic pipeline yielded 190,678 loci containing 266,048 SNPs, which are available on Dryad (https://doi.org/10.5061/dryad.gqnk98t0x), and raw reads and statistics (i.e., call rate, polymorphic information content, heterozygosity, read depth, and reproducibility for all loci and individuals) are accessible from Diversity Array Technology Pty. Ltd., Canberra, Australia (ReportDLitho24‐9512).

Using the package dartR (Gruber et al. 2018), these SNP data were filtered further (in the following order) by call rate (removing loci with more than 10% missing data), minor allele frequencies (removing loci with minor allele frequency lower than 0.005), reproducibility (keeping SNPs present in 100% of replicates), read depth (removing loci with < 5× coverage), removing monomorphic loci, and keeping only one SNP per locus, resulting in 62,477 SNPs in the filtered dataset.

### 2.4 Phylogeny

We inferred a phylogenetic tree using the concatenated sequence tags (the sequences containing the SNPs). We used ModelFinder (Kalyaanamoorthy et al. 2017) to find the optimal substitution model according to the Bayesian Information Criterion (BIC). This was a TVM model with empirical base frequencies and a FreeRate model (Soubrier et al. 2012) with two categories (TVM + F + R2). We inferred the maximum likelihood tree and 1000 ultrafast bootstraps using IQ‐Tree (Minh et al. 2020). An additional time‐calibrated species tree of the most inclusive clade containing all three *Rana* species in this study was adapted from Portik et al. (2023) using the package phytools version 2.4–4 (Revell 2024). We used the “getMRCA” function of phytools to trim the tree to the clade generated by the most recent common ancestor of *R. areolata* , *R. palustris* , and *R. sphenocephala* , to determine the positioning of these species in the context of related taxa and estimate divergence times from one another.

### 2.5 Population Structure

Analyses of population structure were performed on the SNP dataset containing all individuals. First, we estimated ancestry coefficients using sNMF (Frichot et al. 2014), a computationally efficient but accurate method on the basis of sparse nonnegative matrix factorization and least squares optimization. We ran the analyses with the LEA package version 3.16.0 (Frichot and François 2015). We tested 18 combinations of the regularization (α) and tolerance (ε) parameters, choosing the combination with the lowest cross‐entropy. In these test runs, we performed 10 repetitions for each value of K (number of populations) between one and six. We then performed 100 repetitions for each of the values of K with the best combination of α (10,000) and ε (0.0001). We obtained the optimal value of K and individual ancestry coefficients from the run with the lowest cross‐entropy. We also inferred an approximation of population structure by performing principal coordinates analysis (PCoA) with the “gl.pcoa” function of dartR.

### 2.6 Hybridization

We used two programs to detect hybridization in our dataset: NewHybrids (Anderson and Thompson 2002) and the package triangulaR version 1.2.1 (Wiens and Colella 2024). For each program, two parental lineages were compared per run ( *R. areolata* against *R. palustris* or *R. sphenocephala* , respectively, with the inclusion of the relevant interspecific hybrid) with parental reference states identified through the PCoA and sNMF clustering. For NewHybrids, we selected a subset of 200 loci that were the most informative in assessing hybridization, namely loci that showed fixed differences between the parental populations, using the “AvgPic” method in the “gl.nhybrids” function of dartR version 2.9.7. We ran NewHybrids on these datasets for 60,000 sweeps, with the first 10,000 discarded as burn‐in, and repeated this six times for each dataset to confirm convergence of assignment probabilities. For triangulaR, we assigned parental classes for each of the two runs to *R. areolata* and *R. palustris* or *R. sphenocephala* , respectively, and set the allele difference frequency threshold to 0.9 for calculation of the hybrid index.

### 2.7 Breeding Phenology

To assess overlapping interspecific breeding phenology, entry and exit dates for *R. areolata* , *R. paulustris*, *R. sphenocephala* , and hybrids were determined by tracking immigration and emigration to and from the breeding pond for all individuals throughout the duration of fieldwork (encapsulating the entire breeding period for *R. areolata* ). An individual frog was considered to be immigrating to the breeding pond when captured in a pitfall trap on the outside of the drift fence and considered to be emigrating when captured in a pitfall trap on the inside of the drift fence. For each individual frog exiting or entering the pond, the capture time, date, and location around the pond were recorded.

## 3 Results

### 3.1 Cranial Morphometrics

**FIGURE 4:**
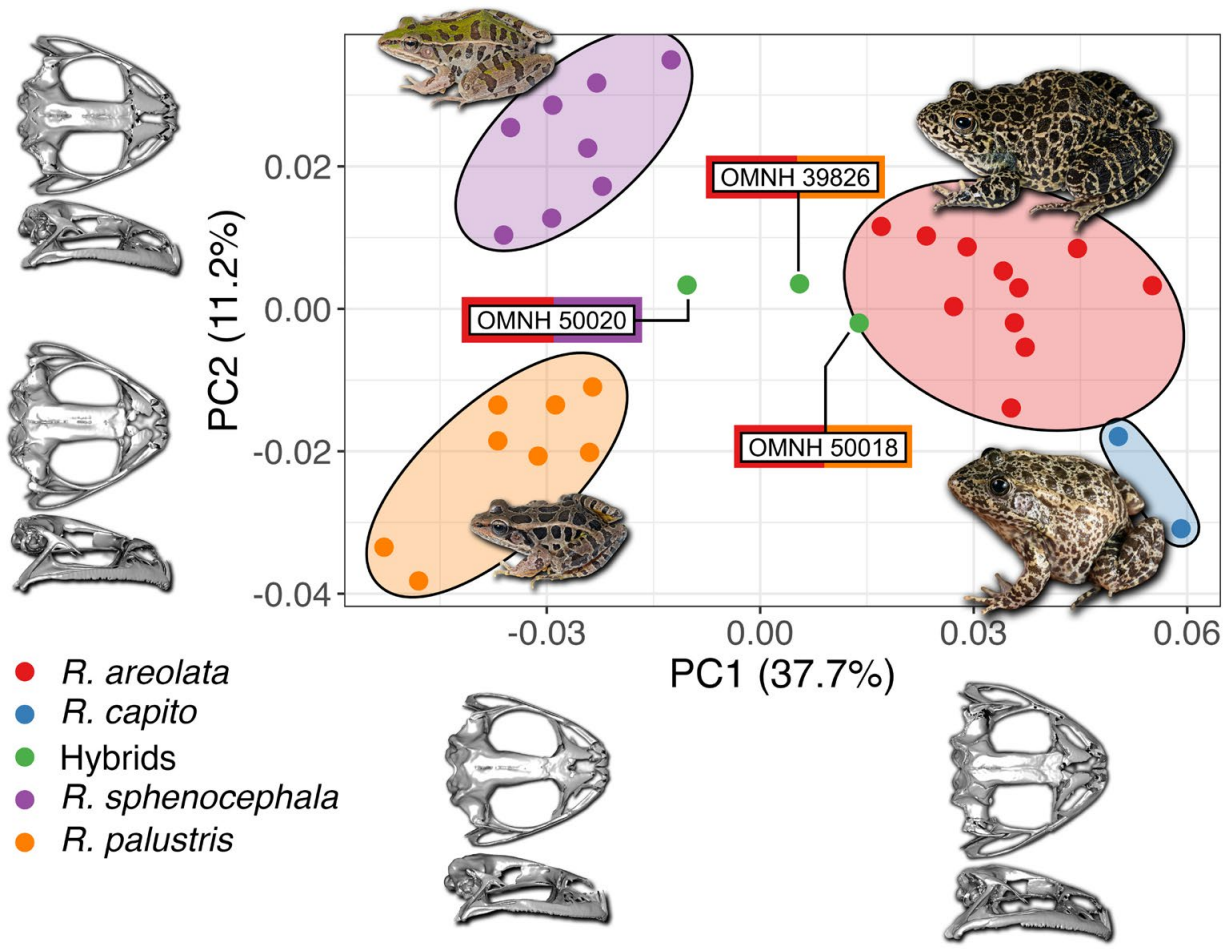
Principal component analysis (PCA) of skull shape variation (on the basis of 35 landmark points) of individuals of four *Rana* species and three hybrids, with each color depicting a different group. Note the intermediate positioning of all three hybrids in PCA space. Ellipses represent the minimum distribution of each species in the PCA space. For visualization, skull configurations on the basis of 3D meshes represent positive and negative landmark transformations associated with each principal component in both dorsal and lateral views. Inset photos depict the crawfish frog ( *R. areolata* ), pickerel frog ( *R. palustris* ), and southern leopard frog ( *R. sphenocephala* ) from the study pond in Atoka Co., Oklahoma (taken by OME) and gopher frog ( *R. capito;* taken by Todd Pierson).

The PCA of the morphological dataset on the basis of cranial landmarks demonstrates that each species differs in skull morphology (Figure 4). The first two PCs explain 48.9% of total skull shape variation and can differentiate all four species. PC1 quantifies the overall dorsal contrast between broader and more robust skulls from *R. areolata* and *R. capito* (positive scores), and narrower and more gracile skulls from *R. sphenocephala* and *R. palustris* (negative scores). PC2 quantifies a dorsal contrast in snout shape, with a rounded snout (U‐shaped; on the basis of curvature of maxilla towards anterior) from *R. sphenocephala* (positive scores) and pointed snout (V‐shaped; on the basis of straightness of maxilla towards anterior) from *R. palustris* and *R. capito* , with *R. areolata* showing intermediate scores. In addition to dorsal features, PC2 also captures lateral differences: towards positive values, skulls appear less curved at both posterior and anterior extremes, maintaining a flatter profile. Toward negative values, however, skulls are more curved at these extremes, contributing to a more convex profile. Other notable morphological variations along PC2 include elongation of the squamosals toward negative values and dilation of the nasals (dorsal view) toward positive values. All three hybrids in morphospace show almost perfect intermediate placement with respect to all parental species (Figure 4), indicating intermediate skull shape in these three individuals.

The np‐MANOVA revealed significant morphological differences among species (*F* = 86.191, *p* < 0.001, *R* ^2^ = 0.9118; see post hoc pairwise comparisons in Appendix 3), indicating that 91.18% of the variation in cranial morphology is attributable to species groupings. The LDA achieved a perfect classification accuracy of 100% through LCV, indicating that cranial morphology provides highly distinctive separation among the species groups (Appendix 4). Individual hybrid classification probabilities showed a high probability of OMNH 39826 and OMNH 50018 belonging to *R. areolata* (Pr > 0.99), whereas OMNH 50020 was not reliably classified as any of its parental species (Table 1).

**TABLE 1:** Posterior probabilities of hybrid individuals belonging to each pure species group on the basis of Linear Discriminant Analysis of skull morphology.

Hybrid ID	Pr ( *R. areolata* )	Pr ( *R. sphenocephala* )	Pr ( *R. palustris* )
OMNH 50018	0.99999	0.00000	0.00001
OMNH 50020	0.0014	0.43096	0.56762
OMNH 39826	0.99452	0.00098	0.00450

*Note:* Probabilities (Pr) indicate the likelihood of each hybrid belonging to species *R. areolata* , *R. sphenocephala* , or *R. palustris* .

### 3.2 Phylogeny

The phylogeny created using concatenated sequence tags provided a robust view of the evolutionary relationships within our dataset, with all major nodes in the tree being accompanied by high support values (100% bootstrap support) (Appendix 5). *Rana sphenocephala* occupies the most distant position relative to the other two species, and little substructure is present within this clade in our phylogeny. A single clade with minimal structure is reconstructed for all *R. areolata* samples, with minimal genetic divergences across the sampled individuals despite a considerable geographic spread for these samples. *Rana palustris* also comprises a single clade, but there are two deep subclades that correspond to a split between populations in the Ozark Plateau and Ouachita Mountains. Although all major nodes in the tree are strongly supported, support values are more variable for recent nodes depicting relationships among individuals within each clade (46%–100% bootstrap support). The two hybrids are positioned intermediate to parental clades, with OMNH 50018 situated intermediate to the *R. areolata* and *R. palustris* clades and OMNH 50020 situated intermediate to the *R. areolata* and *R. sphenocephala* clades (100% bootstrap support).

### 3.3 Population Structure and Admixture

**FIGURE 5:**
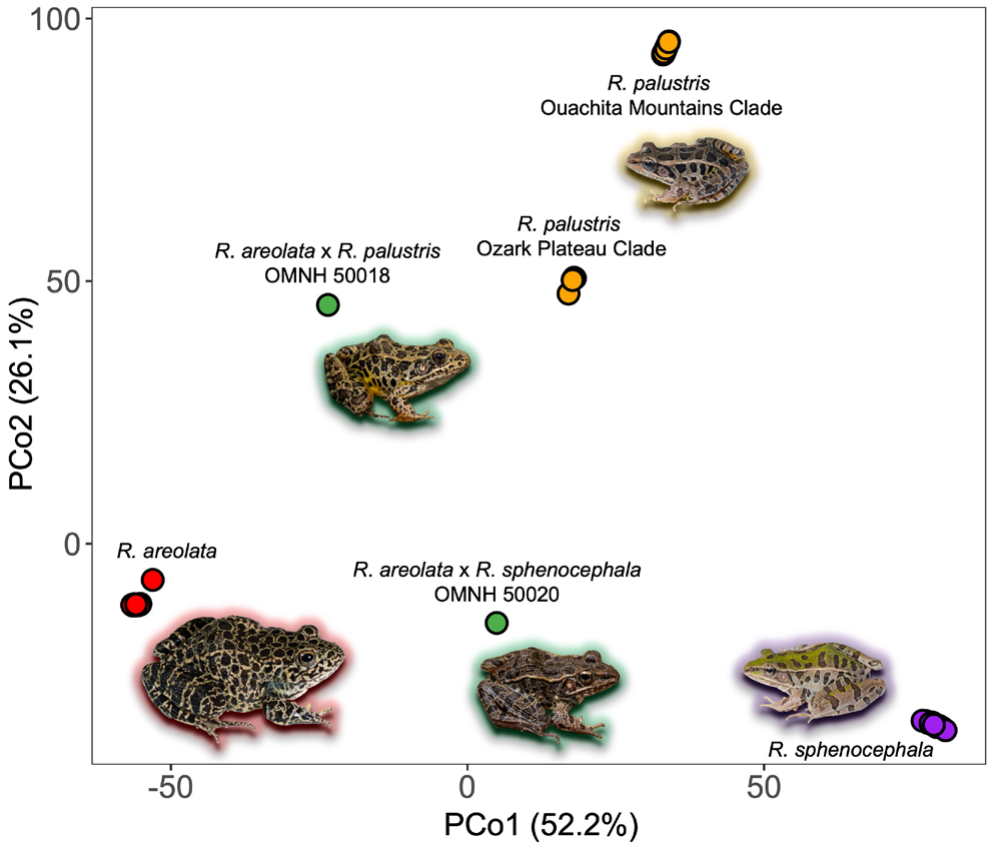
Principal coordinates analysis (PCoA) of genetic distance on the basis of the filtered SNP dataset. Note the intermediate placement of hybrid frogs between parental populations.

The PCoA (Figure 5) indicates the presence of four distinct clusters among the sampled individuals, corresponding to groupings of *R. areolata* , two groups of *R. palustris* (Ouachita Mountains and Ozark Mountains populations), and *R. sphenocephala* . The two hybrid individuals are intermediate to these clusters, with OMNH 50018 being positioned approximately halfway between the *R. areolata* and *R. palustris* (Ouachita Mountains) clusters, and OMNH 50020 approximately halfway between the *R. areolata* and *R. sphenocephala* clusters.

**FIGURE 6:**
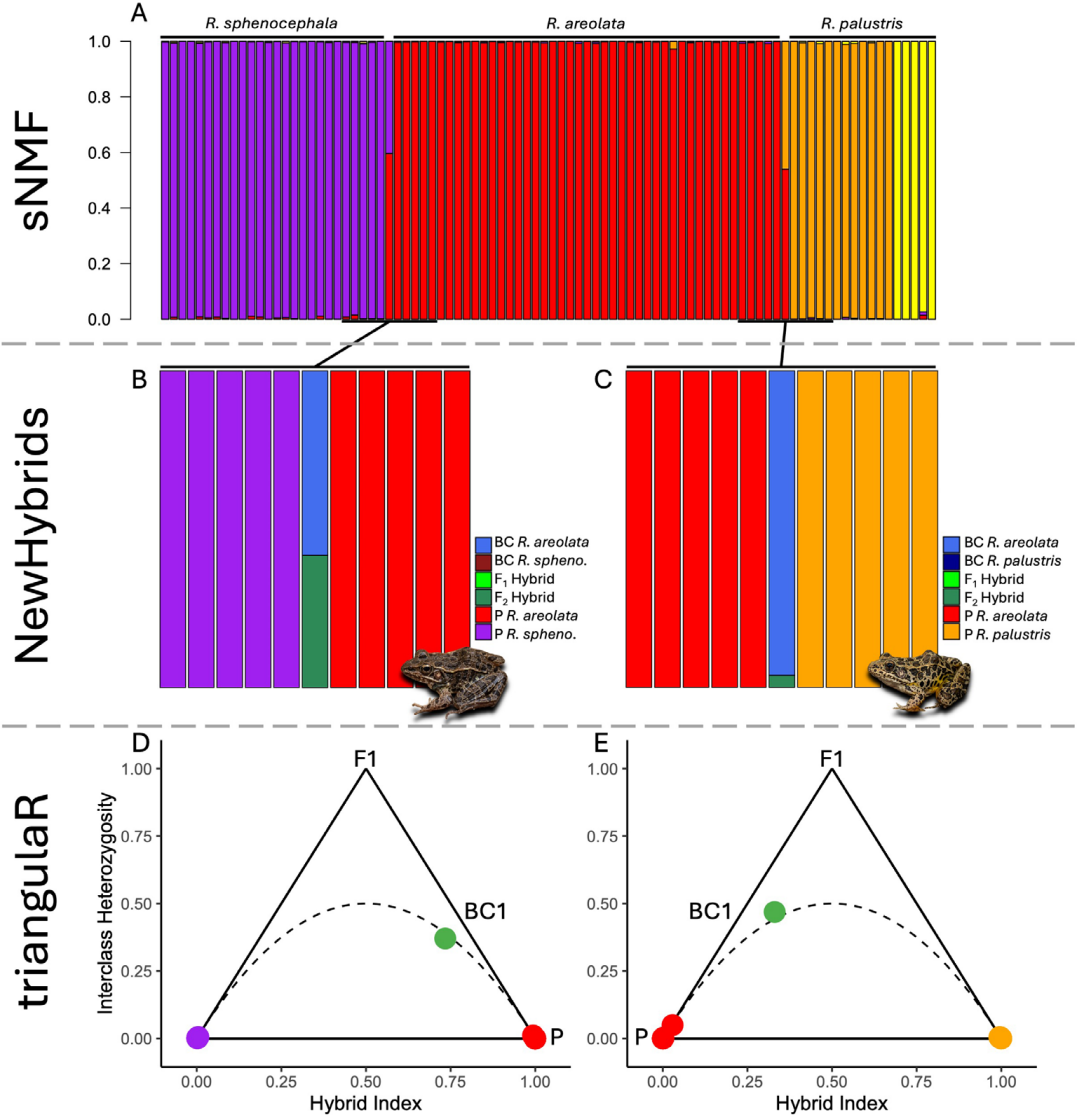
Ancestry coefficients (A) sorted by clade for the three *Rana* spp. and two hybrids (K = 4). Note that the two hybrids have split ancestry coefficients from parental species despite the overall lack of mixed ancestry across the other sampled individuals, though several individuals throughout show low amounts of admixture. NewHybrids (B, C) and triangulaR (D, E) analyses for *R. areolata* × *R. sphenocephala* and *R. areolata* × *R. palustris* indicate a high likelihood of both individuals being F1 × *R. areolata* backcrosses, whereas all other individuals throughout the dataset were identified as belonging to parental populations. It is important to note that (B, C) show bar plots that represent classification probabilities of assignment to specific hybrid categories: F_1_, F_2_, or backcrosses (BC) and are not directly proportional to the sNMF values depicted in (A).

These same general patterns were detected in the sNMF admixture analysis (Figure 6A). At K = 4 (value with the greatest support), our dataset was segmented into four clusters that corresponded to those recovered in the PCoA: *R. areolata* , *R. palustris* “Ouachita Mountains”, *R. palustris* “Ozark Plateau”, and *R. sphenocephala* . The two hybrid individuals are recovered as having mixed ancestry from two parental populations each, with OMNH 50018 having split ancestry coefficients from *R. areolata* and *R. palustris* “Ouachita Mountains”, and OMNH 50020 having split ancestry coefficients from *R. areolata* and *R. sphenocephala* . Minor amounts of admixture (< 5% of ancestry assignment) are present throughout the dataset, but only the two hybrid samples possess major ancestry coefficient contributions from two clusters.

### 3.4 Hybrid Detection

Our analysis conducted in NewHybrids confirmed the hybrid status of both hybrid individuals (Figure 6B,C). OMNH 50018 had a 96.2% likelihood of being a *R. areolata* × *R. palustris* backcrossed into *R. areolata* and a 3.8% likelihood of being an F2 *R. areolata* × *R. palustris* cross. OMNH 50020 had a 58.2% likelihood of being a *R. areolata* × *R. sphenocephala* backcrossed into *R. areolata* and a 41.8% likelihood of being an F2 *R. areolata* × *R. sphenocephala* cross. All other individuals in the dataset were identified as belonging to parental populations of their respective species (99.9% likelihood).

Analysis in triangulaR (Figure 6D,E) identified both OMNH 50018 and OMNH 50020 as first generation backcrosses (bc1; *R. areolata* × *R. palustris* or *R. areolata* × *R. sphenocephala* backcrossed into *R. areolata* , respectively). All other individuals were assigned to parental populations (P1 or P2) apart from a single *R. areolata* (OMNH 50015) with a small amount of *R. palustris* ancestry, as identified in the sNMF analysis. The triangulaR analysis suggests that this individual is beyond four generations backcrossed (bc4) but is not assignable to the pure parental (P1) population.

### 3.5 Breeding Phenology

**FIGURE 7:**
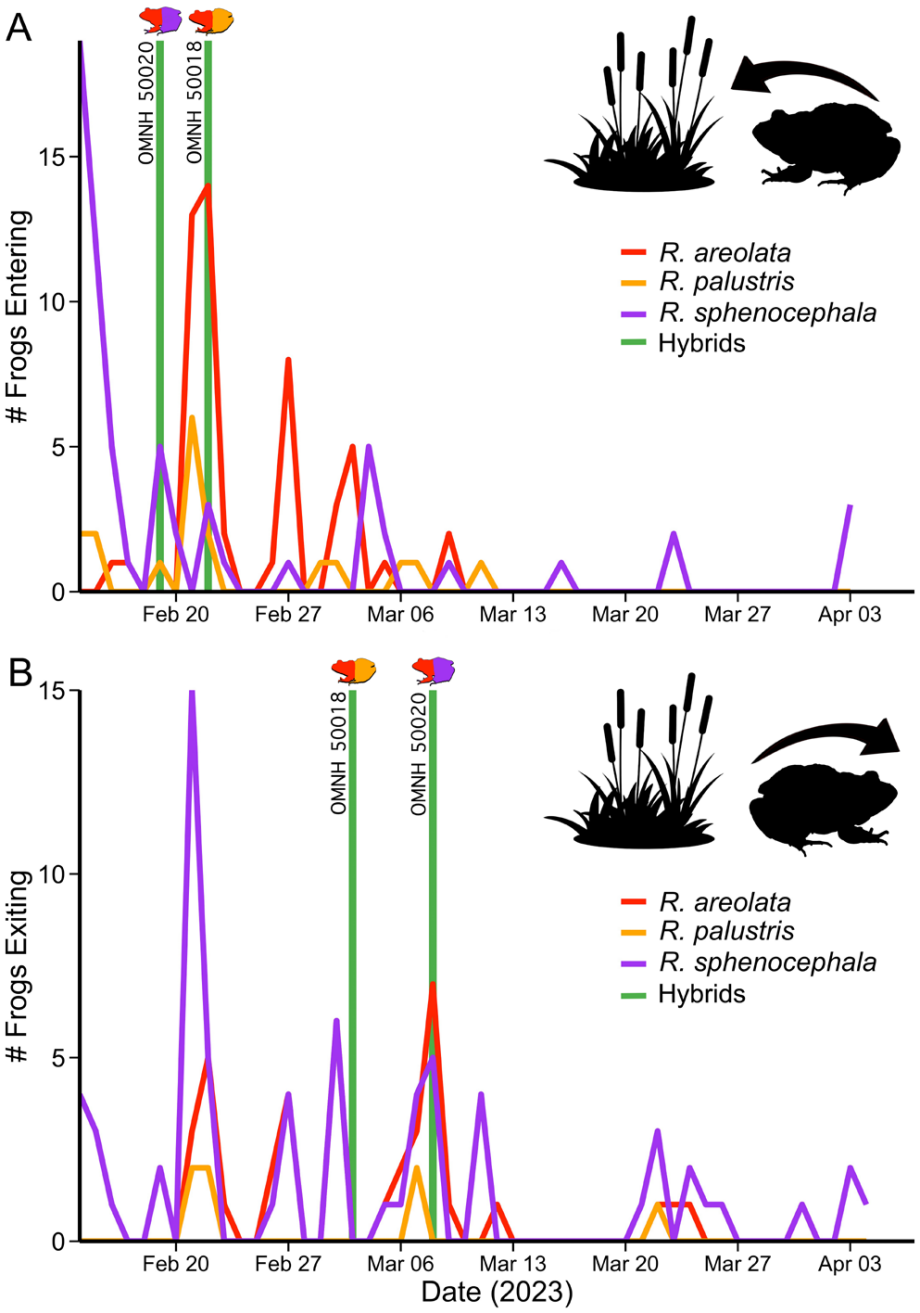
(A) Entry and (B) exit dates for all frogs using the breeding pond in Atoka County, Oklahoma, with entry and exit dates indicated for the two hybrids.

At our study pond, *R. areolata* , *R. palustris* , and *R. sphenocephala* generally showed overlapping immigration and emigration patterns throughout the monitoring period (Figure 7A,B); however, there were slight differences among species. Compared to *R. areolata* and *R. palustris* , which both had a brief movement period of ~28 days, *R. sphenocephala* movement occurred during the entire duration of the study. Immigration for both hybrid individuals corresponded closely to the peak immigration date for *R. areolata* captures. However, *R. areolata* × *R. palustris* (OMNH 50018) did not exit the pond within the emigration dates of either parental species, whereas *R. areolata* × *R. sphenocephala* (OMNH 50020) exited the pond during the peak emigration date for *R. areolata* , which also corresponded to minor activity peaks for both *R. sphenocephala* and *R. palustris* .

## 4 Discussion

### 4.1 Interspecific Hybridization Is Not Uncommon

**FIGURE 8:**
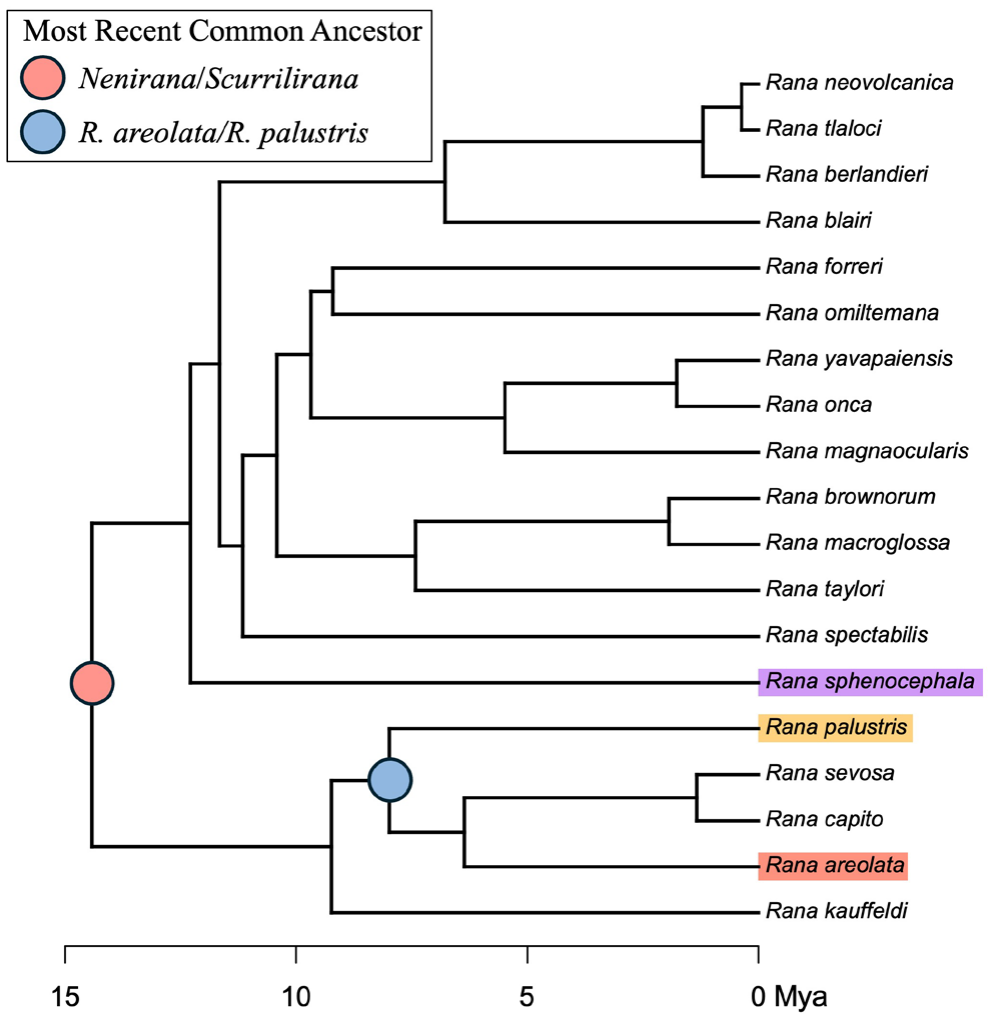
Time‐calibrated species tree adapted from Portik et al. (2023) indicating phylogenetic position of the crawfish frog ( *Rana areolata* ), pickerel frog ( *R. palustris* ), and southern leopard frog ( *R. sphenocephala* ). Divergence times are indicated for splits between *Nenirana* and *Scurrilirana* clades and *R. areolata* and *R. palustris* .

Congeneric taxa that co‐occur over large parts of their geographic distributions are generally expected to have considerable barriers in place (both pre‐ and post‐zygotic) to inhibit widespread hybridization, which, if unrestricted, would erode the divergence between lineages. Despite this expectation, natural hybridization appears to be abundant across the tree of life and has been documented across diverse taxa and various ecological scenarios (Taylor and Larson 2019). In frogs, hybridization has been characterized for both sympatric and parapatric species distributions (Austin et al. 2011; Peek et al. 2019), in taxa that diverge in complex courtship behavior (Nali et al. 2023), and even species with differing ploidies (Gerhardt et al. 1994; Haddad et al. 1994). Our study shows that across the 43 individuals of *R. areolata* we captured during the first year of a population study (Edwards et al. 2025), at least two are backcrossed hybrids: one exhibiting split ancestry with *R. sphenocephala* and the other with *R. palustris* , suggesting hybridization is presently occurring and involves multiple species, which are remarkably distantly related (Figure 8). Moreover, through a morphological analysis of a specimen collected in 2003, we find strong evidence for a third hybrid. We demonstrate that natural hybridization is not a rare occurrence and that distant taxa on independent evolutionary trajectories for as long as 15 My remain reproductively compatible.

Nevertheless, consistent with the expectation of broadly sympatric congeners having strong barriers in place to limit hybridization, we see little evidence of widespread admixture dynamics in the *R. areolata* samples within our data set (Figure 6A), with only a single individual displaying admixture from the co‐occurring population of *R. palustris* (Figure 6A; Ouachita Mountains clade). Although these results suggest that some hybrid individuals may be viable and capable of reproduction, they also indicate that admixture is only present in negligible amounts across these three species.

### 4.2 Ecological Dimensions of Hybridization

Misdirected amplexus in anurans is geographically widespread and is currently known from at least 180 species (Serrano et al. 2022; Soni et al. 2024). Unsurprisingly, overlapping breeding phenology among heterospecifics is one of the main drivers influencing the prevalence of this behavior. Misdirected amplexus is more frequently reported among explosive breeders than among prolonged breeders and is more common in temperate regions where seasonal climate fluctuations confine breeding to brief temporal periods than in tropical regions where reproductive periods may be under reduced timing constraints (Soni et al. 2024). Although *R. sphenocephala* and *R. palustris* are generally characterized as prolonged breeders (Given 2005; Saenz et al. 2006), *R. areolata* exhibits an explosive breeding strategy because of its limited breeding window (Terrell et al. 2023). Although each species has unique advertisement calls (Engbrecht et al. 2015; Lannoo et al. 2018; Banks et al. 2024), they overlap in breeding phenology (Figure 7), occur in temperate regions (Figure 2), and have male‐biased operational sex ratios (Hardy and Raymond 1991; Kinney 2011; Lannoo and Stiles 2020). Together, these characteristics likely amplify levels of scramble male–male competition for mates (Wells 2007) and dampen the ability of females to recognize and differentiate conspecific mating signals and exhibit mate choice, especially when these signals are overlapping in time with those of other taxa. At our study pond in 2024, we documented a single misdirected amplexus event between a female *R. areolata* and a male *R. sphenocephala* (Edwards et al. 2024), suggesting that hybrids are likely the result of overlapping breeding strategies and competitive dynamics.

Both prezygotic and postzygotic reproductive isolation increase with divergence time between taxa (Sasa et al. 1998), and prezygotic isolation evolves faster than postzygotic isolation in sympatric species (Coyne and Orr 1989, 1997; Sasa et al. 1998). With these expectations in mind, we might expect *R. areolata* to have a greater propensity for hybridization with *R. palustris* (5–10 Ma divergence) than with *R. sphenocephala* (10–15 Ma divergence), given its more recent divergence from the former (Portik et al. 2023; Figure 8). Despite this expectation, we found hybrids between both of these species and *R. areolata* (Figure 6). In some cases, hybridization occurs between highly divergent taxa, with examples documented for vertebrate lineages separated by as long as 21 million years, even longer than the divergence between our study taxa (Prager and Wilson 1975).

Among the species studied here, prezygotic isolation in comparison to postzygotic isolation is likely weaker, as evidenced by frequent misdirected amplexus (Serrano et al. 2022), implying that behavioral isolation through species‐specific mate recognition may be insufficient to fully prevent hybridization, even among highly diverged taxa as examined in this study. Similar breakdowns in behavioral isolation have been observed in other anurans, such as *Pseudacris* (Lemmon and Juenger 2017) and *Hyla* species (Gerhardt et al. 1994), where overlapping calls or relaxed female preference (Abt and Reyer 1992) can permit hybridization. Furthermore, the explosive‐like breeding strategy of *R. areolata* may reduce the effectiveness of call‐based mate discrimination, increasing opportunities for heterospecific encounters compared to species with prolonged breeding seasons. Postzygotic isolation mechanisms, however, may still significantly constrain gene flow. Although we detected hybrids, the limited number and the predominance of backcrossed rather than F1 individuals may suggest potential intrinsic barriers such as reduced hybrid viability or fertility as observed in other anuran systems (Sherman et al. 2010; Parris et al. 1999). In addition, sexual selection may act against hybrids if hybrid male acoustic signals fail to attract mates (Lemmon and Lemmon 2010) or if hybrid female mating preferences do not align with the calls of either parental species (Schmidt and Pfennig 2015), thereby reducing reproductive success despite viability and fertility. Nonetheless, the presence of backcrossed individuals indicates that some hybrid offspring are capable of surviving and reproducing, though postzygotic costs may restrict long‐term introgression. Future research on hybrid fitness, reproductive success, and acoustic behavior will be essential to clarify the strength and directionality of reproductive isolation in this system.

### 4.3 Hybrid Frogs Are Diagnosable by Morphology

Although both of the hybrids we found in the field were genetically confirmed as *R. areolata* backcrosses (Figure 6), their cranial morphology was intermediate to that of parental populations (Figure 4). However, in a statistical framework, the classification probabilities for OMNH 50018 and OMNH 39826 on the basis of the LDA model strongly supported assignments to the *R. areolata* cluster (Table 1), consistent with these two individuals being positioned at the periphery of the discriminant space of *R. areolata* (Appendix 4). This suggests that the skull morphology of *R. areolata* × *R. palustris* when backcrossed with *R. areolata* , more closely resembles *R. areolata* , while still retaining some morphological features of *R. palustris* . The other hybrid individual, OMNH 50020, identified as *R. areolata* × *R. sphenocephala* backcrossed with *R. areolata* , had nearly equal likelihoods of belonging to *R. sphenocephala* and *R. palustris* on the basis of classification probability (Table 1) and was positioned precisely at the midpoint among parental groups in discriminant space (Appendix 4). Therefore, we conclude that hybrids in this system, including backcrosses, can be effectively diagnosed by their intermediate cranial morphology.

Morphology alone is often a poor indicator of parentage in anuran hybrids because of high phenotypic variation, particularly at the F1 generation (Lamb and Avise 1987), and hybrids frequently exhibit overlapping traits with one or both parental species (Lodé and Pagano 2000; Majtyka et al. 2022; Nali et al. 2023). Exemplifying this, a study of hybridization in the morphologically similar sister taxa, *Hyla cinerea* and *H. gratiosa* , found that nearly 40% of genetically verified hybrids would have been misclassified as pure parental species on the basis of morphometrics alone, and that 25% of backcrossed individuals were indistinguishable from F1 hybrids (Lamb and Avise 1987), illustrating the challenge of using morphological data (Watters et al. 2016) in the absence of genetic verification. However, probably because of the major interspecific differences in head morphology among our focal taxa (Engbrecht et al. 2011), our micro‐CT‐based morphological analysis was effective in distinguishing hybrid individuals, revealing clear intermediate cranial morphologies that aligned with genetic classifications.

### 4.4 Conservation and Population Dynamics

*Rana areolata* is a species of conservation concern throughout its distribution, with recent range‐wide declines attributable to landscape change and habitat loss (Engbrecht et al. 2012; Kross and Willson 2022; Boycott et al. 2024). In other amphibians, genetic pollution has been implicated as a major threat, particularly with respect to species introductions (Fitzpatrick and Shaffer 2007), though limited examples of naturally occurring hybridization threats have been characterized (Austin et al. 2011). In the most severe cases, genetic swamping can occur when a rare species interbreeds with a more abundant one, or outbreeding depression can occur when unique genetic adaptations are lost because of genetic incompatibilities (Allendorf et al. 2001; Roberts et al. 2010).

Because of broad sympatry among *R. areolata* , *R. sphenocephala* , and *R. palustris* , hybridization has the potential to occur across a large area of co‐occurrence for all three species (see areas of sympatry in Figure 2). Given the environmental pressures affecting *R. areolata* across much of its distribution (Lannoo and Stiles 2020), ongoing genetic testing and population monitoring in vulnerable areas would facilitate the identification of potential conservation concerns if hybridization occurs elsewhere. Population declines alone can cause heightened rates of hybridization because of decreased abundance of one species; these rates may further be exacerbated if habitat modification is present (Gottelli et al. 1994; Gutiérrez et al. 2007; Todesco et al. 2016). Our results indicate very low levels of introgression among *R. areolata* , *R. sphenocephala* , and *R. palustris* , suggesting that hybridization is unlikely to be of conservation concern in Oklahoma, but the possibility remains that hybridization could potentially pose a risk where *R. areolata* exists in small or isolated populations (Templeton 1986).

### 4.5 Deep Genetic Structure in *Rana palustris* in the Interior Highlands

Our results point to the presence of deep genetic structure in *R. palustris* between the two sampled regions included in our dataset. In the ordination of genetic distances (Figure 5), *R. palustris* forms two distinct clusters with clear separation from one another, and that also differ from the *R. areolata* and *R. sphenocephala* clusters. Additionally, two deep subclades were recovered in the phylogenetic analysis (Appendix 5) and ancestry analyses (Figure 6A) indicated an overall lack of admixture between these two lineages.

Although *R. palustris* has an expansive geographic distribution across eastern North America (Figure 2), its distribution at finer scales may be tied to geological formations, particularly at its western limits (Resetarits Jr. and Aldridge 1988). These two lineages have apparently been isolated from one another by the impermeability of the Arkoma Basin barrier, because of their dependence on caves and other subterranean habitats associated with the Interior Highlands (Resetarits Jr. and Aldridge 1988). The two clades recovered herein are associated with two areas in the Interior Highlands Region: the Ozark Plateau and Ouachita Mountains. Despite the geographic proximity of these two areas, the Ozark Plateau and Ouachita Mountains have differing geological histories and relatively distinct faunal affinities, particularly with respect to amphibian taxa (Martin et al. 2016). The Ozarks and Ouachitas were formed by disparate geological processes during the Pennsylvanian (Thornbury 1965). The two highland regions have remained disjunct since their formation, separated by the lowland habitat of the Arkoma Basin (Carlton and Cox 1990), which would have limited dispersal for amphibian taxa associated with upland habitats. The biogeographical history of plethodontid salamanders in the Interior Highlands suggests a pattern of colonization where species in the Ozark Plateau share close affinities to the Eastern Highlands, whereas species in the Ouachita Mountains share closer affinities to those in the Coastal Plains and Edwards Plateau (Martin et al. 2016). Coupled with our genetic data, these patterns suggest a plausible scenario for two independent colonizations of *R. palustris* into the Interior Highlands Region, with frogs in the Ozark Plateau representing a colonization event from the Eastern Highlands, whereas those from the Ouachita Mountains represent a colonization event from the Coastal Plains.

## 5 Conclusion

Our study integrated genomic, morphological, and phenological data to explore and assess hybridization among three broadly sympatric ranid frogs. We provide the first evidence of natural hybridization of *R. areolata* , with not only a close congener ( *R. palustris* ) but also with a considerably more divergent species ( *R. sphenocephala* ). Two lines of evidence indicate that both collected hybrids were backcrossed individuals: *R. areolata* × ( *R. areolata* × *R. sphenocephala* ) and *R. areolata* × ( *R. areolata* × *R. palustris* ). We also determined that hybrids are likely capable of producing viable offspring, as indicated by low levels of introgression in many other sampled *R. areolata* . Thus, infrequent hybridization may be a normal occurrence in this system, but it does not appear to affect the genetic integrity of any of the taxa. However, the frequency of hybridization and its regional or range‐wide impacts on gene flow are unknown in other areas where these species co‐occur. Future work should aim to understand the fitness consequences of hybrid offspring, evaluate the prevalence of hybridization across space and time, conduct a clade‐level appraisal of hybridization in the family Ranidae, and identify the factors that lead to hybridization among more than two species.

## Author Contributions

**Owen M. Edwards:** conceptualization (equal), data curation (equal), formal analysis (equal), investigation (equal), methodology (equal), writing – original draft (equal), writing – review and editing (equal). **Neil R. Balchan:** conceptualization (equal), data curation (equal), formal analysis (equal), investigation (equal), methodology (equal), writing – original draft (equal), writing – review and editing (equal). **Kaleb M. Banks:** data curation (supporting). **Bo Zhang:** funding acquisition (equal), writing – review and editing (equal). **Fabio A. Machado:** formal analysis (supporting), supervision (supporting), writing – review and editing (equal). **Michael S. Reichert:** funding acquisition (lead), resources (equal), supervision (supporting), writing – review and editing (equal). **Damien Esquerré:** formal analysis (supporting), methodology (supporting), project administration (equal), resources (supporting), supervision (lead), validation (equal), writing – review and editing (equal).

## Ethics Statement

All work conducted in this study followed approved Oklahoma State University Institutional Animal Care and Use Committee protocols (AUP #22‐56 and #22‐33), and voucher specimens were collected under Letter of Authorization from the Oklahoma Department of Wildlife Conservation (W2308) or Scientific Collectors Permits issued to N.R.B. (10372246) or O.M.E. (10794524).

## Conflicts of Interest

The authors declare no conflicts of interest.

## Data Availability

Computed tomography data (tiff stacks and mesh files) are available on MorphoSource (Project ID 000720563: https://www.morphosource.org/projects/000720563?locale=en). Data and scripts for all morphological and genomic analyses are available on the Dryad Digital Repository (https://doi.org/10.5061/dryad.gqnk98t0x).

## Appendix 1

### Examined Specimens (Cranial Morphometrics)

*Rana areolata* : Atoka County, Oklahoma; OMNH 39111, 39112, 39228, 39470, 39471, 42015, 42017, 42021, 42024, 42025, 50032. *Rana areolata* × *Rana palustris* : Atoka County, Oklahoma; OMNH 39826, 50018. *Rana areolata* × *Rana sphenocephala* : Atoka County, Oklahoma; OMNH 50020. *Rana capito* : Lee County, Florida; OSUS A499, A514. *Rana palustris* : Atoka County, Oklahoma; OMNH 50010, 50011, 50016, 50017, 50021, 50023, 50026, 50031. *Rana sphenocephala* : Atoka County, Oklahoma; OMNH 50009, 50012–50014, 50019, 50022, 50024, 50025.

*OMNH, Sam Noble Oklahoma Museum of Natural History; OSUS, Oklahoma State University Collection of Vertebrates.

### Sequenced Tissues (Genomic SNPs)

*Rana areolata* : Atoka County, Oklahoma; OMNH 50015, 50032, 50048–50050, CF 44, 53, 54, 57–61, 68–70, 72–76, 80, 82, 83. Coal County, Oklahoma; OMNH 50027, 50029, 50037. Johnston County, Oklahoma; FHSM 18258. Latimer County, Oklahoma; OMNH 50033. Mayes County, Oklahoma; OMNH 50044, 50045. McIntosh County, Oklahoma; OMNH 50035. Nowata County, Oklahoma; OMNH 50041. Okfuskee County, Oklahoma; OMNH 49663. Okmulgee County, Oklahoma; OMNH 50036. Pittsburg County, Oklahoma; OMNH 50030. Pontotoc County, Oklahoma; FHSM 18239. Rogers County, Oklahoma; OMNH 50038–50040, 50052. Tulsa County, Oklahoma; OMNH 50042, 50043, 50047. Wagoner County, Oklahoma; OMNH 50034. Washington County, Oklahoma; OMNH 50056. *Rana areolata* × *Rana palustris* : Atoka County, Oklahoma; OMNH 50018. *Rana areolata* × *Rana sphenocephala* : Atoka County, Oklahoma; OMNH 50020. *Rana palustris* : Atoka County, Oklahoma; OMNH 50010, 50011, 50016, 50017, 50021, 50023, 50031. Delaware County, Oklahoma; OMNH 46578, 46580, 46583, 46585, 46587. LeFlore County, Oklahoma; OCRG 2685, OMNH 41724, 42049, 43457, 43600. *Rana sphenocephala* : Atoka County, Oklahoma; OMNH 50009, 50012–50014, 50019, 50022, 50024, 50025, 50028. Haskell County, Oklahoma; OMNH 50054 (batch of 5). Lincoln County, Oklahoma; OMNH 50055 (batch of 5). Payne County, Oklahoma; OMNH 50053 (batch of 5). Payne County, Oklahoma; OMNH 50248–50253.

*CF, Crawfish Frog Population Monitoring Field Series; FHSM, Fort Hays Sternberg Museum of Natural History; OCGR, Oklahoma Collections of Genomic Resources; OMNH, Sam Noble Oklahoma Museum of Natural History.

## Appendix 2

Specimen photographs of individual hybrids examined in this study. From left to right: Male *Rana areolata* × *R. sphenocephala* (OMNH 50020), female *R. areolata* × *R. palustris* (OMNH 50018), and female *R. areolata* × *R. palustris* (OMNH 39826) that was discovered at the Sam Noble Oklahoma Museum of Natural History (OMNH).

## Appendix 3

Results of np‐MANOVA post hoc pairwise comparisons between species on the basis of cranial morphology. All comparisons between species were statistically significant at *p* < 0.05.

**Table:**

Species comparison	*F*‐statistic	*R* ^2^	*p*
*R. palustris* vs. *R. areolata*	136.4	0.889	< 0.001
*R. palustris* vs. *R. sphenocephala*	46.4	0.768	< 0.001
*R. palustris* vs. *R. capito*	65.1	0.890	0.021
*R. areolata* vs. *R. sphenocephala*	123.5	0.879	< 0.001
*R. areolata* vs. *R. capito*	12.3	0.528	0.018
*R. sphenocephala* vs. *R. capito*	103.8	0.929	0.025

## Appendix 4

Distribution of individual specimens and species averages on the first two axes of the linear discriminant analysis of skull shape variation among ranid species. Colored ellipses represent the 95% confidence intervals on the basis of the pooled within‐group covariance of the a priori defined species groups. LD1 and LD2 are the two linear discriminants that best separate the species, highlighting distinct clustering for each species and the intermediate positioning of hybrids.

## Appendix 5

Phylogenetic tree including all sampled individuals estimated using concatenated sequence tags. Note the overall lack of sequence divergence within the *Rana areolata* and *R. sphenocephala* clades, but the presence of two divergent subclades within the *R. palustris* clade.
